# HIV epidemic among men who have sex with men in the Czech Republic, 2016: high time for targeted action

**DOI:** 10.2807/1560-7917.ES.2017.22.48.17-00079

**Published:** 2017-11-30

**Authors:** Viktor Mravčík, Michal Pitoňák, Robert Hejzák, Barbara Janíková, Ivo Procházka

**Affiliations:** 1 National Monitoring Centre for Drugs and Addiction, Office of the Government of the Czech Republic, Prague, Czech Republic; 2 Department of Addictology, First Faculty of Medicine, Charles University and General Teaching Hospital, Prague, Czech Republic; 3 National Institute of Mental Health, Klecany, Czech Republic; 4 Czech AIDS Help Society, Prague, Czech Republic; 5 Institute of Sexology, First Faculty of Medicine, Charles University, Prague, Czech Republic

**Keywords:** HIV/AIDS, MSM, PrEP, stigma

## Abstract

Reported incidence of human immunodeficiency virus (HIV) infection in the Czech Republic increased steeply over the past decade from 90 new cases in 2005 to 266 in 2015. This increase is almost exclusively attributed to sexual transmissions between men who have sex with men (MSM). In 2015, there were 79% (n=210) newly diagnosed cases among MSM, 17% (n=45) were attributed to heterosexual transmission and 1% (n=3) to people who inject drugs. Interventions targeted at MSM have not yet been prioritised in the broadly focused national HIV prevention strategy which this is envisaged to change in the programme set out for 2018 to 2022. The national budget for HIV prevention has been reduced, however, and this remains. Availability of voluntary counselling and testing has decreased substantially in the past decade. Post- and pre-exposure prophylaxis for sexual intercourse among MSM are not part of the HIV prevention policy and the concept of treatment as prevention is not fully recognised. Provision of a combined prevention strategy with a focus on MSM, reflecting the above factors including stigmatisation, should contribute to reverse the development of a concentrated HIV epidemic among MSM in the Czech Republic.

## Introduction

Infection caused by human immunodeficiency virus (HIV) remains substantial public health concern worldwide associated with high mortality and morbidity, low quality of life, reduced life expectancy and high treatment costs, affecting certain vulnerable communities and subpopulations disproportionately [1].

Despite public health measures, significant HIV transmission continues in Europe. In 2015, 153,407 people were newly diagnosed with HIV in the World Health Organization (WHO) European Region (98,177 of them in Russia), which corresponds to 17.6 newly diagnosed infections per 100,000 of population (7.6 without Russia). In European Union and European Economic Area (EU/EEA) countries, the number of newly diagnosed infections reached 6.3 per 100,000 population; the lowest rate (2.3 per 100,000 population) was reported from central Europe. Sex between men was the most frequent route of transmission in the EU/EEA (42.2%), while the proportion of people who inject drugs (PWID) was small (4.2%). Whereas heterosexual and PWID transmission decreased over the last decade in the majority of EU/EEA countries, the number of newly reported HIV infections among men who have sex with men (MSM) has continuously increased [2].

## Situation in the Czech Republic

The Czech Republic with its 266 newly-diagnosed HIV cases in 2015 (2.5 per 100,000 population) ranks, together with Slovakia and Slovenia, among the EU/EEA countries with the lowest reported incidence. At the same time, the Czech Republic, together with Croatia, Hungary and Slovenia, is one of the EU/EEA countries with the highest proportion of MSM among newly-diagnosed cases (approx. 80% and over) in the region [2].

**Figure 1:**
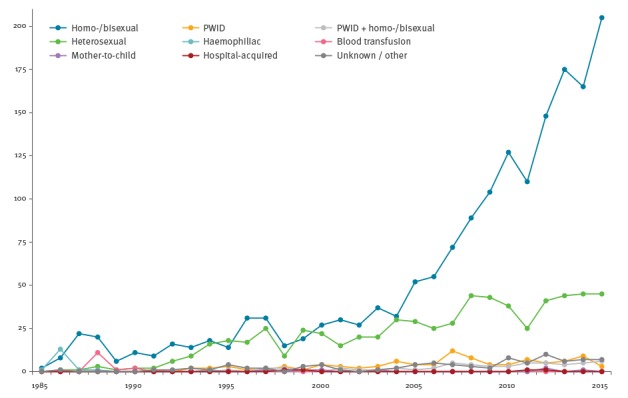
Annual number of newly diagnosed HIV infections among Czech citizens and residents, by transmission category, Czech Republic, 1985–2015

HIV: human immunodeficiency virus; PWID: people who inject drugs.

In the past decade, the number of newly diagnosed HIV infections has been growing rapidly from 90 cases in 2005 to 266 cases annually in 2015. The increase has been attributed exclusively to sexual transmission between men; other modes of transmission have shown stable (PWID) or slightly increasing (heterosexual transmission) rates (Figure 1). In 2015, there were 210 (79%) newly diagnosed cases among MSM, 45 cases (17%) were attributed to heterosexual transmission and three cases (1%) to PWID. More than half of the newly diagnosed cases (51% in 2015) lived in the capital city, Prague [3], while in the general population, Prague inhabitants represent 12% of the population of the Czech Republic. Of 266 newly diagnosed HIV infections in 2015, 46 (17%) cases were diagnosed late (acquired immunodeficiency syndrome (AIDS) or symptomatic non-AIDS stage) and 85/237 (36%) had CD4^+^ T-cell counts < 350/mL at diagnosis [3].

Source of the data: Malý et al. [3].

In a bio-behavioural survey among MSM carried out in six European cities (Barcelona, Bratislava, Bucharest, Ljubljana, Prague and Verona) in 2008 and 2009 as part of the Sialon project (the project for capacity building in HIV/syphilis prevalence estimation using non-invasive methods among MSM in southern and eastern Europe), HIV testing was performed among 400 MSM in Prague and resulted in a seroprevalence rate of 2.6%, which was the lowest among the participating cities [4]. However, this rate does not necessarily account for the dynamics of the HIV epidemic in the Czech Republic in recent years. Up to the end of 2016, the cumulative number of newly-diagnosed HIV infections among MSM reached nearly 2,000 cases [3,5], with at least 1,000 of them resident in Prague. In addition, many younger homo- or bisexual people are registered at the permanent address of their parents, but in fact they live, work or study in Prague. After we included undiagnosed cases, based on a rather conservative estimate of 30% latency (Němeček and Malý estimated up to 47% undiagnosed HIV cases in the Czech Republic in 2014 [6]; recent data for the EU/EEA estimated 17%, however, the estimate for the Czech Republic is not available [7]), a total of 1,500 HIV-positive MSM were estimated in Prague. Applying results from a study in England [8], we estimate that at least 5% of Prague’s male population are MSM and thus, at least 26,000 MSM aged 15 years and older can be assumed to live in Prague, of whom 1,500 are HIV-positive. This would correspond to an HIV seroprevalence of more than 5% among MSM in Prague, which is according to the WHO a threshold for a concentrated HIV epidemic.

Little is known about behavioural factors contributing to recent HIV transmission among MSM in the Czech Republic because research in this field is lacking. Data from the Sialon project showed lower condom use among Czech MSM during sexual intercourse with steady and especially with casual partners, compared with MSM from other participating countries; only 29.0% of the Czech MSM used condoms in the last anal intercourse with a male partner [4]. The latest European MSM Internet Survey (EMIS) data returned higher rates of condom use among Czech MSM (41.0% during the last anal intercourse), however, this rate was lower than in other European countries. Nevertheless, the rate of unprotected anal intercourse with casual partners in the last 12 months (35.8%) was comparable with the EU median (39.8%), and casual sex as such in the last 12 months was less frequently reported in the Czech Republic (63.2%, the lowest rate in EU) than in the EU (75.1%) [9].

According to the only available online survey that focused on chemsex primarily among MSM in the Czech Republic in 2016 (n = 948, among them 710 reported gay, 143 bisexual and 11 lesbian sexual orientation), methamphetamine was used during sexual activities by 14%, ecstasy by 12%, gamma-hydroxybutyric acid (GHB)/gamma-butyrolactone (GBL) by 8%, mephedrone by 3% and other drugs (likely alcohol, cannabis or poppers) by 15% of respondents in the last 12 months (data not shown). Recent use of drugs characteristic for chemsex has been found much less prevalent among MSM in Prague than for example in London, Amsterdam or Paris, but comparable with many other metropolitan cities in Europe [10].

In addition to behavioral factors, further we discuss important structural factors moderating recent HIV epidemic among MSM in the Czech Republic.

## HIV/AIDS prevention policy and its funding

Nowadays, so-called combination prevention, consisting of individual interventions such as testing, including community-based testing, with rapid tests and self-testing, behavioural risk reduction, condom distribution, needle and syringe programmes, treatment of other sexually transmitted infections (STI), and antiretroviral medications in treatment and preventive contexts, is recommended at the national and local levels, with sufficient scale, combination of interventions and coverage of the most-affected population groups including MSM [11].

The basic strategy document in the field of HIV/AIDS in the Czech Republic is the National programme for tackling HIV/AIDS in the period from 2013 to 2017 [12]. Although sex between men is the main transmission route and MSM are the most affected at-risk group in the Czech Republic, neither the MSM group nor activities targeting sex between men are defined as priorities and there is no single specific activity focusing on MSM in the programme. For example, the MSM group is listed without any emphasis as one among the 18 target groups of the programme. The next HIV/AIDS national programme for the period from 2018 to 2022, which is now being drafted, is expected to explicitly prioritise activities targeting MSM.

The main financial mechanism for HIV prevention in the Czech Republic is the grant system of the Ministry of Health for the National programme for tackling HIV/AIDS, which is launched every year. As the grant programme runs on an annual basis, it might be a flexible tool for adjusting the policy priorities according to the actual situation, however, the same terms of reference for the grant call have been maintained over the last decade with no emphasis on addressing the main transmission route of the HIV epidemic – sex between men [13].

**Figure 2:**
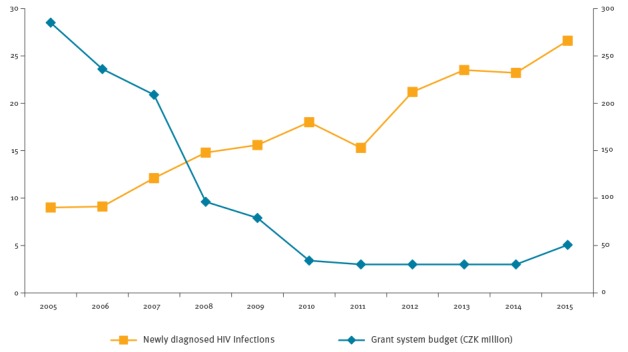
Annual budget of the grant system of the National programme for tackling HIV/AIDS and newly diagnosed HIV infections in the Czech Republic in 2005–2015

AIDS: acquired immunodeficiency syndrome; HIV: human immunodeficiency virus.

In contrast to the increasing number of newly diagnosed HIV infections, the prevention budget for the grant system of the National programme for tackling HIV/AIDS has been rapidly decreasing in the past decade. While in 2005, the budget was CZK 28.5 million (ca EUR 950,000), it was reduced in 2015 to CZK 5.1 million (ca EUR 190,000) (Figure 2). Although this HIV prevention budget could already be viewed as modest in 2005, it was indeed insufficient in 2015 even though since the beginning of the new millennium, the antiretroviral therapy (ART) treatment, which is available in eight regional HIV/AIDS treatment centres in the Czech Republic, has been covered by the public health insurance and is not included in the HIV prevention budget anymore. It shall be noted that volunteer work in HIV/AIDS prevention is not common in the Czech Republic and involvement of target groups’ peers and volunteers is therefore limited. In comparison, the harm reduction services for PWID, financed from the budget on drug policy, are supported with CZK 200 million annually (ca EUR 7.5 million), which is approximately 40 times more [14].

The exchange rate was approximately CZK 30 for EUR 1 in 2005 and CZK 27 for EUR 1 in 2015.

The competition for resources is tough between various state and non-governmental organisations in the field of HIV prevention, and in the absence of programmatic prioritisation, activities focused on MSM are not preferentially supported. Analysis of the titles of the projects supported in 2016 showed that among 37 projects financed with CZK 9.8 million in total, only eight projects with a summary budget of CZK 3 million (31%) focused specifically on provision of testing, condoms, outreach work or social assistance to MSM [15].

The policy development in the area of HIV has to be seen in the context of overall public health policy. The public health service (consisting of public health authorities and public health institutes) underwent negative development in the period 2006 to 2012, leading to a substantial reduction of its capacity (e.g. 43% reduction of personnel capacity) especially in the fields of public health protection, health monitoring or health promotion [16]. This reduction concerned also the network of HIV/AIDS counselling centres operated within the public health service: while there were 54 of these centres run by the public health service in 2006 with broad national coverage, there were 23 centres in 2014, of which only 13 were run by the public health service. Consequently, the access to voluntary (anonymous) counselling and testing (VCT) decreased substantially [16], even though the voluntary testing and the testing of contacts of HIV-positive cases show the highest proportions of case detection, 0.7% and 4.3%, respectively [3]. Availability and accessibility of VCT for MSM is crucial for early detection of HIV infection, especially when analysis of CD4^+^ T cells at diagnosis shows that MSM have the lowest proportion of late diagnoses among all transmission groups in EU; this demonstrates higher testing rates and awareness in the MSM community compared with other at-risk populations [2].

## Prophylactic use of antiretroviral medications

Besides post-exposure prophylaxis (PEP), antiretroviral medications have recently been recommended for pre-exposure prophylaxis (PrEP). PrEP is recommended especially in HIV-negative MSM and transgender individuals at high risk of HIV acquisition [17]. Continuous and on-demand PrEP has demonstrated high efficacy in preventing HIV seroconversion among MSM: it was well tolerated, the evolution of HIV resistance was low, while compensatory increased sexual risk behaviour was not observed [18]. PrEP as a standard modality or within demonstration projects is available or planned in 20 European countries, however, the Czech Republic is not among them [19]. Although some evidence suggests caution with respect to increased risk of other STIs among MSM on PrEP, implementation of PrEP can also be viewed as an opportunity for better STI detection and treatment among the most at-risk MSM [20]. However, use of PrEP by individuals infected with HIV but unaware of their infection can lead to the generation and further transmission of resistant viruses [21].

The Czech clinical guidelines for HIV contain a defined algorithm for PEP [22]. In an EMIS survey from 2010 [8], experience with PEP was reported by 0.4% of the MSM in the Czech Republic, which was among the lowest proportions of the countries participating in the survey. However, PrEP is not mentioned in the Czech clinical guidelines and as such is not formally available [22]. There are no data on informal use of PrEP in the Czech Republic, however, there are anecdotal reports from VCT centres on PrEP use among MSM. 

Early initiation of treatment leads to viral supression and decreases or eliminates HIV transmision to sexual partners (treatment as prevention, TasP). Regarding ART initiation, the recent clinical guidelines, in place since 2016, recommend start of treatment with CD4^+^ T cell counts > 500/mL also in asymptomatic infections [22]. The previous version of the clinical guidelines adopted in 2012 recommended start at threshold CD4^+^ T cells < 350/mL or CD4^+^ T cells = 350–500/mL if other factors (such as dramatic CD4^+^ T cell decrease, high viral load, co-infections, other comorbidities, sex with a steady HIV-negative partner or pregnancy) were present; a threshold of CD4^+^ T cells > 500/mL was previously recommended only ‘*in special circumstances*’ [23]. Recent data on continuum of care indicate that 71% of those with diagnosed HIV infection receive ART, and 85% of them have viral suppression [6].

## Stigma affecting MSM

Structural stigma refers to societal conditions, norms or policies that compromise the opportunities, resources and well-being of a socially marginalised group such as sexual minorities [24]. A growing body of research demonstrates that structural stigma in particular serves as a key driver of poor health outcomes among sexual minorities [25]. HIV infection in MSM represents double stigma on a personal and a community level associated with social exclusion, discrimination, rejection and violence [26].

In the Czech Republic, prejudice related to sexual minorities has been documented [27]. Also sex between men has been framed mostly negatively in some of the Czech mainstream media. Qualitative analysis of Czech media reports about HIV/AIDS showed that HIV transmission was often presented as a ‘*punishment for an abnormal behaviour such as injecting drug use or homosexual orientation*’ [28].

In 2016, stigmatisation of HIV-positive MSM in the Czech Republic has become a topic of public discussion in the aftermath of the prosecution of 30 HIV-positive homosexual men, most of them on ART treatment, who allegedly violated the Criminal Code for spreading infectious human disease practicing condomless sex [29,30]. Criminalisation for the reckless transmission of HIV is an intrinsic part of the structural stigma and remains controversial since HIV risk can increase with criminalisation as it reinforces stigma and fear of HIV-positive people and undermines HIV prevention efforts [31-34].

## Conclusions and recommendations

MSM represent the group most at risk of HIV infection in the Czech Republic. The number of newly diagnosed HIV infections among MSM has grown rapidly in recent years and is currently reaching the level of a concentrated epidemic, especially in the capital city, Prague.

However, in the authors’ opinion, the current epidemiological situation is not reflected in national policy development. Neither the core strategic and programmatic documents nor the financial support in the field of HIV/AIDS prevention prioritise activities focusing on MSM. Despite the current state of the HIV epidemic, HIV/AIDS prevention is underfinanced and is not focussed on specific activities targeting MSM such as outreach programmes, strategy for destigmatisation, condom distribution, community testing and early identification of possible contacts, or social support. PrEP has not been implemented or piloted so far and PrEP or TasP are not yet fully recognised by public health authorities or clinicians [35]. In addition, prejudice and stigma, together with other societal and structural factors, influences the disproportionate incidence of HIV in MSM.

Finally, we underline the importance of evidence-based formulation of priorities in prevention strategy and its funding schemes in order to achieve better provision of an HIV/AIDS integrated prevention strategy including PrEP, recognition of TasP and destigmatising HIV/AIDS and MSM.
